# Supplementary material for: A Comparison between Conventional and Extracorporeal Cardiopulmonary Resuscitation in Out-of-Hospital Cardiac Arrest: A Systematic Review and Meta-Analysis
Source: Healthcare (Basel). 2022 Mar 21;10(3):591. doi: 10.3390/healthcare10030591 (PMC8955421; doi:10.3390/healthcare10030591)
Supplement: Supplementary file 1 [file healthcare-10-00591-s001.zip › healthcare-1590531-supplementary.pdf]

Supplementary Meterials

Year, first author	Point	Lower limit	Upper limit	p-Value	eCPR	CPR	Relative weight
2013 Maekawa	4.53	0.83	24.65	0.08	7 / 24	2 / 24	6.14
2014 Kim	5.99	1.60	22.49	0.01	15 / 76	3 / 76	11.09
2014 Sakamoto	4.84	2.31	10.16	0.00	47 / 336	9 / 270	19.98
2016 Choi 2009-2013	3.12	1.44	6.76	0.00	76 / 656	28 / 590	29.81
2016 Choi 2011-2015	3.63	1.69	7.80	0.00	79 / 666	30 / 640	35.18
2017 Schober	3.38	1.75	6.54	0.00	80 / 673	43 / 872	39.95
2017 Yannopoulos	3.39	2.05	5.61	0.00	106 / 735	69 / 1042	49.62
2019 Nakashima	3.51	2.25	5.49	0.00	135 / 985	73 / 1199	57.91
2019 Patricio	3.18	2.08	4.86	0.00	147 / 1065	81 / 1279	66.59
2020 Bougouin	2.84	1.59	5.08	0.00	191 / 1590	1221 / 13945	77.07
2020 Kitada	2.31	1.21	4.40	0.01	212 / 1897	1608 / 16223	87.27
2020 Yannapoulos	2.49	1.32	4.70	0.00	218 / 1911	1609 / 16238	91.81
2020 Yoshida	2.84	1.50	5.40	0.00	224 / 1949	1618 / 16731	100.00
**All studies**	2.84	1.50	5.40	0.00			

Cumulative statistics / Cumulative odds ratio (95% CI)

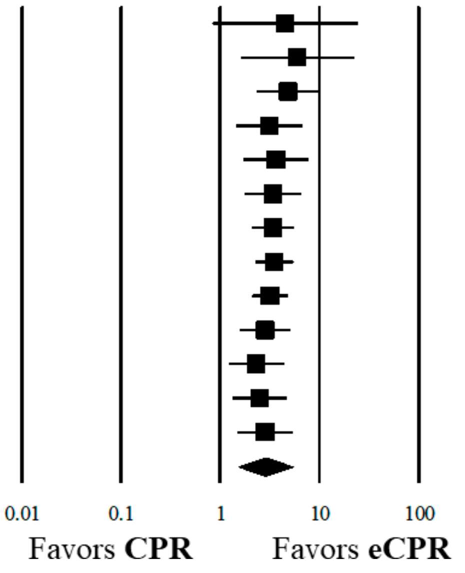

Figure S1. Cumulative statistical analysis for any favorable outcome

Year, first author	Point	Lower limit	Upper limit	p-Value
2013 Maekawa	2.76	1.42	5.36	0.00
2014 Kim	2.67	1.39	5.13	0.00
2014 Sakamoto	2.72	1.39	5.31	0.00
2016 Choi 2009-2013	3.10	1.51	6.40	0.00
2016 Choi 2011-2015	2.63	1.37	5.05	0.00
2017 Schober	2.85	1.47	5.53	0.00
2017 Yannopoulos	2.73	1.40	5.34	0.00
2019 Nakashima	2.69	1.39	5.24	0.00
2019 Patricio	3.04	1.52	6.07	0.00
2020 Bougouin	3.37	1.55	7.32	0.00
2020 Kitada	3.45	1.92	6.21	0.00
2020 Yannapoulos	2.67	1.39	5.12	0.00
2020 Yoshida	2.49	1.32	4.70	0.00
**All studies**	2.84	1.50	5.40	0.00

Statistics with study removed / Odds ratio (95% CI) with study removed

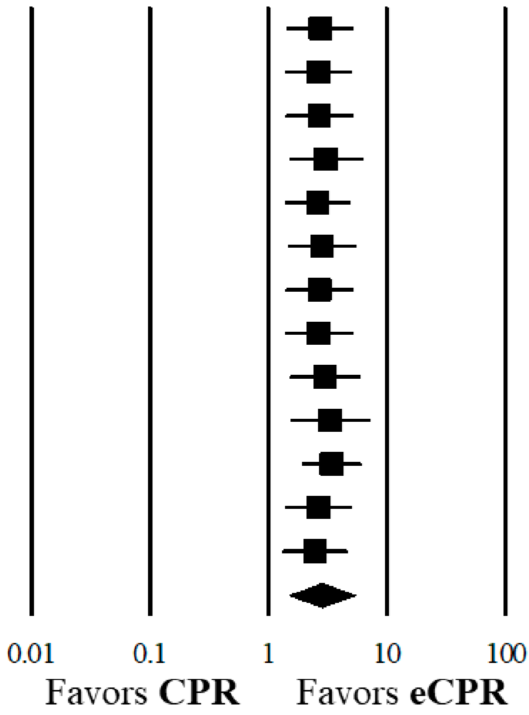

Figure S2. Sensitivity analysis for composite outcome of any favorable outcome
